# Base-Modified Nucleosides: Etheno Derivatives

**DOI:** 10.3389/fchem.2016.00019

**Published:** 2016-04-28

**Authors:** Zofia Jahnz-Wechmann, Grzegorz R. Framski, Piotr A. Januszczyk, Jerzy Boryski

**Affiliations:** Institute of Bioorganic Chemistry, Polish Academy of SciencesPoznan, Poland

**Keywords:** nucleoside analogs, ethenonucleosides, wyosine, fused heterocycles, tricyclic acyclovir, antiviral activity

## Abstract

This review presents synthesis and chemistry of nucleoside analogs, possessing an additional fused, heterocyclic ring of the “etheno” type, such as 1,N^6^-ethenoadenosine, 1,N^4^-ethenocytidine, 1,N^2^-ethenoguanosine, and other related derivatives. Formation of ethenonucleosides, in the presence of α-halocarbonyl reagents and their mechanism, stability, and degradation, reactions of substitution and transglycosylation, as well as their application in the nucleoside synthesis, have been described. Some of the discussed compounds may be applied as chemotherapeutic agents in antiviral and anticancer treatment, acting as pro-nucleosides of already known, biologically active nucleoside analogs.

## Introduction

**Figure 1:**
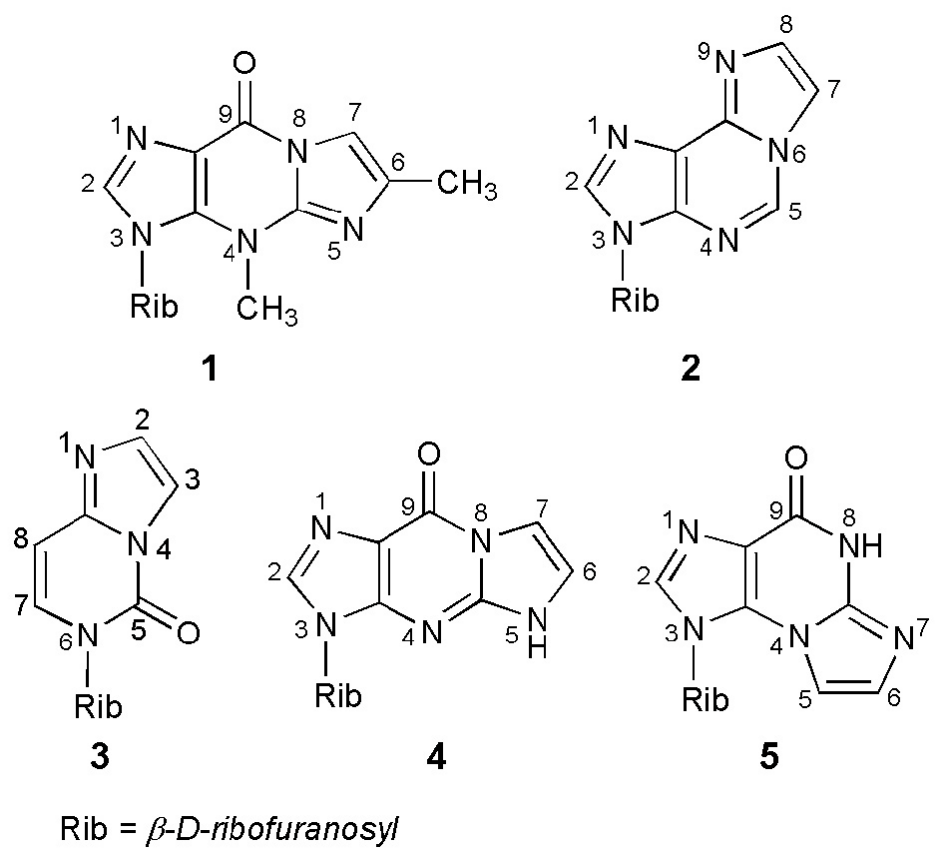
**Naturally occurring wyosine (1) and synthetic ethenonucleosides**.

A number of base-modified nucleosides demonstrate significant biological properties, acting, among others, as antiviral or antitumor agents. One of the possible modifications of the nucleoside is their transformation into ethenonucleosides. Thus, nucleosides or their respective heterocyclic bases, possessing an exocyclic amino group, as well as a neighboring endocyclic nitrogen, can react with some bifunctional reagents (e.g., α-halocarbonyl compounds) to form products with an additional five-membered ring of the imidazole type. The ethenonucleosides basically do not occur in nature; the only exception here are Y-nucleosides, strongly fluorescent tricyclic derivatives of guanosine, occurring in the anticodon loop of some phenylalanine transfer ribonucleic acids (tRNA^Phe^; Blobstein et al., 1975; Kasai et al., 1976). Wyosine, i.e., 3-methyl-1,N^2^-(prop-1-ene-1,2-diyl)guanosine (compound **1**; Figure 1), is the simplest example of the Y-nucleosides family. The other known ethenonucleosides, such as 1,N^6^-ethenoadenosine (**2**), 1,N^4^-ethenocytidine (**3**), 1,N^2^-ethenoguanosine (**4**), or N^2^,3-ethenoguanosine (**5**), have been obtained *via* chemical modification of parent ribonucleosides.

In the following review, we drew our attention mainly to chemistry of ethenonucleosides and related compounds. Their biological properties, fluorescence study, antiviral action, and mutagenic effect on the nucleic acid level have already been presented in other reviews or recent articles (e.g., Secrist et al., 1972; Steiner et al., 1973; Kusmierek and Singer, 1992; Golankiewicz and Ostrowski, 2006; De Clercq, 2010; Gómez-Bombarelli et al., 2011; Jahnz-Wechmann et al., 2015). Therefore, in this review, we will focus on the chemical properties of ethenonucleosides, barely mentioning or shortly discussing their other features.

## Synthesis

### Derivatives of adenosine and cytidine

The first ever synthesis of ethenonucleosides, a new class of chemically modified components of nucleic acids, was reported in the 1970's. Thus, treatment of 9-methyladenine and 1-methylcytidine with chloroacetaldehyde (CAA), and using them as models for appropriate nucleosides, gave previously unknown fluorescent bases (Kochetkov et al., 1971). In turn, a similar reaction of adenosine and cytidine allowed to obtain the respective nucleosidic products—1,N^6^-ethenoadenosine (**2**) and 1,N^4^-ethenocytidine (**3**; Secrist et al., 1972). The optimum pH value for this reaction was established at 3.5 for cytosine, and 4.5 for adenosine (Kochetkov et al., 1971; Barrio et al., 1972;). The yield of the synthesis usually varied from satisfactory to quantitative. They also showed that another, naturally occurring nucleoside–guanosine, fulfilled the structural requirements to form its etheno derivative, however it practically did not react with aq. chloroacetaldehyde (Barrio et al., 1972).

Since that time, a great number of etheno products derived from adenosine or cytidine have been reported (e.g., Steiner et al., 1973; Barrio et al., 1976; Kost and Ivanov, 1980; Sattsangi et al., 1980; Leonard, 1984; Kifli et al., 2004). It has also been shown that not only haloaldehydes and haloketones may be applied in the synthesis of etheno derivatives of A and C. Other reagents of a similar nature are: bromomalonaldehyde (Nair et al., 1984), N-(tert-butoxycarbonyl)-2-bromoacetamide and 2-chloroketenene diethyl acetal (Leonard and Cruickshank, 1985), malonaldehyde (Seto et al., 1985), epoxy carbonyl compounds (Nair and Offerman, 1985), 1-halooxiranes (Guengerich and Raney, 1992), 1,2-dicarbonyl reagents (Shapiro and Hachmann, 1966) mucochloric acid (Mäki et al., 1999), or 3-chloropropyne (Virta et al., 2003). Quite recently, Xie et al. (2014) have reported synthesis of etheno adenosines and cytidines, *via* a copper-catalyzed domino Michael oxidative cross-coupling, with nitroolefins.

**Scheme 1:**
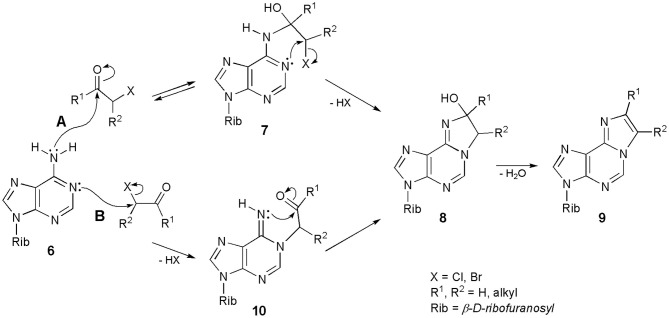
**Two possible mechanisms of synthesis of 1,N^6^-ethenoadenosine and its derivatives**.

Interestingly, after decades of synthesizing ethenonucleosides with the use of α-halocarbonyl reagents, the mechanism of their formation still remains unclear. Regioselectivity of the reaction is not controversial: carbonyl group of CAA reacts with the exocyclic amino group of nucleosides, while chloromethylene portion alkylates the endocyclic nitrogen. This has been demonstrated in the reaction with α-haloketones, possessing an additional alkyl substituent, i.e., α-bromopropionaldehyde and chloroacetone (Krzyzosiak et al., 1983), or with the use of deuterium-labeled reagents (Kost and Ivanov, 1980). However, the sequence of events in the formation of etheno rings is not obvious in the majority of cases. Biernat et al. (1978) and Krzyzosiak et al. (1983) postulated an initial condensation of carbonyl group of the α-halocarbonyl reagents and exocyclic 6-amino group of adenosine (**6;** Scheme 1: mechanism **A**), leading to an unstable intermediate (**7**). The reaction is reversible. Intermediate **7** then undergoes intramolecular cyclization to a stable intermediate **8**, as the result of alkylation of endocyclic N1, and this process is irreversible. Compound **8** may now undergo dehydration to the final 1,N^6^-etheno product (**9**). The intermediate product **8** has been isolated, and its structure confirmed by the ^1^H NMR data (Biernat et al., 1978).

Another, equally convincing pathway of synthesis is presented in Scheme 1; mechanism **B**. Adenosine (**6**) is initially alkylated at the N1-position to furnish an intermediate product (**10**). This step is irreversible, because the N-C bond in 1-alkyladenines is very stable. However, compound **10** immediately undergoes a ring-closure reaction, to produce compound **8** and again, **8** is converted to **9** in the dehydration step. Considering the sequence of events in the formation of 1,N^6^-ethenoadenosine (**2**) and its derivatives (general structure **9**), we generally agree with the previous assumption of Kost and Ivanov (1980) that both mechanisms may take place, depending on the structure of α-halocarbonyl reagent, applied in this transformation. Thus, haloaldehydes and chloroacetaldehyde in particular, can react according to the pathway **A**, while haloketones and 1-halooxiranes (Guengerich and Raney, 1992) would rather react in line with the mechanism **B**. In our opinion, the structure of the starting adenosine precursor has very little or no effect on the discussed process. The structure of substrate, however, will have a deciding effect in the synthesis of ethenoguanosines (see Chapter Derivatives of Guanosine).

The mechanisms presented in Scheme 1 apply to the synthesis of 1,N^4^-ethenocytidine (**3**) as well. Other N-alkyl nucleosides derived from A and C may also react with CAA, e.g., N^6^-isopentenyladenosine (i^6^A), N^6^-methyladenosine (m^6^A), or N^4^-methylcytosine (Barrio et al., 1972; Biernat et al., 1978; Sattsangi et al., 1980). Because of the presence of additional N-alkyl group, the reaction intermediates of the type **8** cannot be dehydrated to fluorescent etheno compounds, and the reaction stops at this stage. Other alkylated substrates, like 1-metyladenosine (m^1^A) or 3-metylcytidine (m^3^Cyt), do not react with CAA and related reagents, due to the absence of amidine system in their structures (Barrio et al., 1972).

### Derivatives of guanosine

**Scheme 2:**
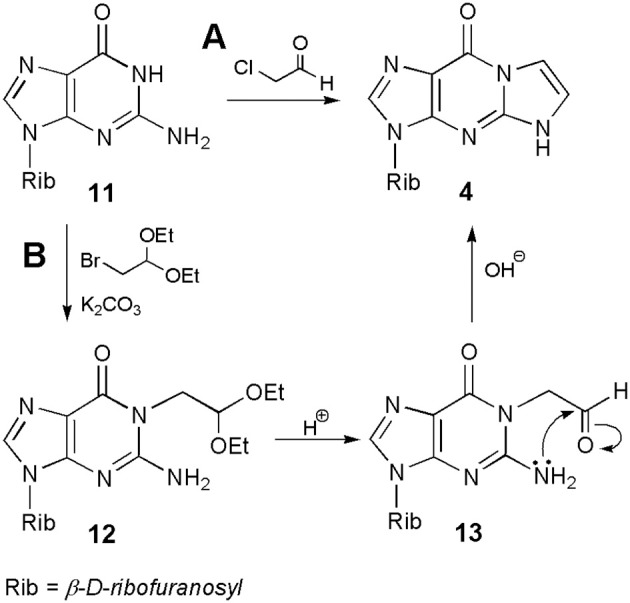
**Synthesis of 1,N^2^-ethenoguanosine**.

Much of the discussion so far concerned the synthesis of ethenonucleosides derived from adenosine and cytidine. As stated above, guanosine (**11**; Scheme 2) is almost unreactive under conditions suitable for modification of A and C, and therefore this nucleoside gained relatively less attention in the beginning. However, as shown in subsequent years, ethenoguanosine and its derivatives deserve special attention due to their biological properties and usefulness in nucleoside chemistry. Because of its structure, guanosine can form two isomeric nucleosides, 1,N^2^-ethenoguanosine (**4**), called “linear” form, and “angular” N^2^,3-etheno compound (**5**). The former compound may be obtained from guanosine under treatment with chloroacetaldehyde, nevertheless the yield is usually very poor, e.g., 7.5% at pH 6.4 after 7 days (Sattsangi et al., 1977). The yield of **4** can be improved at higher pH values, e.g., 13% at pH 9–10, or under anhydrous conditions, by using sodium salt of guanosine prepared in DMSO and 90% ethereal solution of CAA (yield 24%; Boryski, 1990). However, the best yield of the synthesis may be obtained in a two-step procedure. As shown in Scheme 2, alkylation of guanosine (**11**), with bromoacetaldehyde diethyl acetal in the presence of potassium carbonate, leads to the 1-substituted derivative (**12**; O^6^-isomers are usually reaction side-products), which gives an unstable aldehyde intermediates, upon acidic hydrolysis (**13**). The latter compound (corresponding to compound **10** in the synthesis of 1,N^6^-ethenoadenosine) undergoes a spontaneous intramolecular cyclization and dehydration to 1,N^2^-ethenoguanosine (**4**; overall yield 50%; Boryski, 1990; Boryski et al., 1991). In turn, another compound of this type, a 6-hydroxymethyl derivative of 1,N^2^-ethenoguanosine may be obtained in the reaction of guanosine with glycidaldehyde (Nair and Turner, 1984). More recently, it has been shown that a linear ethenoguanosine analog can be synthesized in a satisfactory yield (54%), when applying CAA and 6-chloro-2-aminopurine substrates at 70°C (Horejsi et al., 2006).

**Scheme 3:**
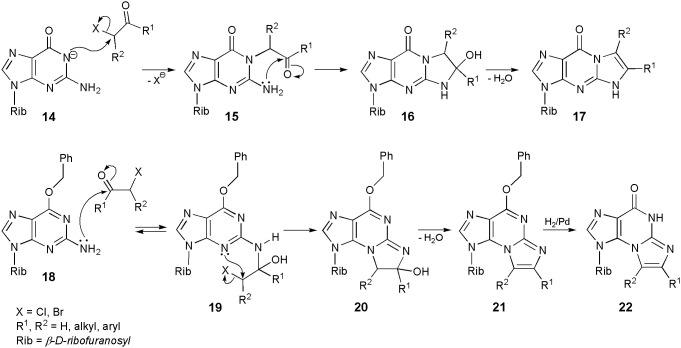
**Mechanisms of synthesis of 1,N^2^-ethenoguanosine, N^2^,3-ethenoguanosine and their derivatives**.

The reactions of guanosine with substituted haloketones are much easier to perform than in the case of chloroacetaldehyde. In a typical procedure (Scheme 3), guanosine is converted to its sodium salt (**14**), which is readily alkylated with haloketones to an unstable intermediate (**15**). Intramolecular cyclization to **16**, followed by dehydration (usually in alkaline media) results in the formation of a linear tricyclic product (general structure **17**; yield 70–90%; Kasai et al., 1976; Golankiewicz and Folkman, 1983). Consequently, the sequence of events is obvious in this case: the initial 1-alkylation is followed by condensation of carbonyl group and precipitation of exocyclic amine residue. However, O^6^-substiution dramatically changes the course of reaction. For example, treatment of O^6^-benzylguanosine with chloroaldehyde gives the angular N^2^,3-ethenoguanosine (**5**; Sattsangi et al., 1977; Kusmierek et al., 1987, 1989). Our proposed mechanism for this modification is depicted in Scheme 3. It is well-known that 3-alkylguanosines may never be obtained by direct intermolecular alkylation, and therefore O^6^-benzylguanosine (**18**) cannot be alkylated with CAA at the 3-position. Instead of that, **18** undergoes an initial condensation with carbonyl group of the aldehyde, what leads to an unstable intermediate (**19**). This process is perhaps reversible, but compound **19** may undergo an intramolecular 3-alkylation as well, forming the tricyclic product (**20**). After dehydration to compound **21**, the O-benzyl substituent can be removed by applying catalytic hydrogenolysis to obtain the final product (general structure **22**; R^1^,R^2^ = H for **5**). Quite similar results were reported in the case of O^6^-ethylguanosine (Kusmierek et al., 1987).

In line with the proposed mechanism, guanosine residue in nucleic acids should be modified by CAA to the linear 1,N^2^-ethenoguanosine (**4**). On the other hand, treatment of nucleic acids with vinyl chloride gave, among other ethenonucleosides, the fluorescent angular product—N^2^,3-ethenoguanosine (**5**). It should be noted at this point that vinyl chloride, a strong carcinogen, is metabolized *in vivo* to chloroacetaldehyde, which reacts with nucleobases. (Kusmierek et al., 1987; Singer et al., 1987). The formation of **5** can be rationalized in the following way: guanosine, participating in the base pairing of double helix, may exist in a tautomeric form, other than that of the free nucleoside, i.e., O^6^=C–N^1^H → HO^6^–C=N^1^. The structure of the second tautomer corresponds to that of the O^6^-alkylguanosine, and this is why N^2^,3-ethenoguanosine is formed at the nucleic acid level.

### Other ethenonucleosides

**Scheme 4:**
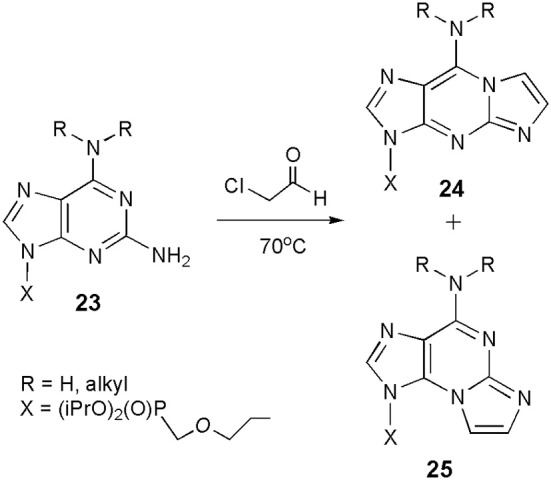
**Formation of the etheno products in the reaction of 2,6-diaminopurine nucleosides with CAA**.

Compounds related to both forms of ethenoguanosine may also be synthesized in the reaction of CAA with 2,6-diaminopurine nucleosides (general structure **23**; Scheme 4), which are considerably more reactive than guanosine (Horejsi et al., 2006). Interestingly, the reaction leads to a mixture of linear (**24**) and angular (**25**) products. Their ratio clearly depends on substitution in the N^6^-position. As reported, unsubstituted substrate (R=H, H) gave a 1:3 mixture of **24** and **25**, N^6^-cyclopropyl derivative was exclusively transformed to the linear product (**24**), whereas modification of N^6^,N^6^-diethyl compound yielded only the angular isomer (**25**). So far, we cannot find any rational explanation for those unexpected results. Moreover, 2,6-diaminopurine nucleosides may be considered as analogs of both adenosine and guanosine, and if so, one could expect rather the formation of products structurally related to 1,N^6^-ethenoadenosine (**2**).

However, in general, the synthesis of ethenonucleosides proceeds with high selectivity. The sugar moiety has no substantial effect on the course and yield of this modification. Therefore, the etheno derivatives can be obtained from a variety of protected and unprotected substrates, including e.g., ribo- and deoxyribonucleosides, arabinofuranosides, acyclonucleosides, their phosphates, cyclic phosphates and polynucleotide chains of RNA and DNA.

## Chemistry

As far as the sugar portion is concerned, ethenonucleosides are quite similar in their reactivity to other nucleosides. Therefore, in the present review we shall focus our attention on reactions involving the aglycon moiety of these etheno-bidged compounds and their derivatives.

### Alkylation

Some intensive studies on alkylation of ethenonucleosides have been performed in the case of guanosine derivatives. The reason for that was a close structural similarity of 1,N^2^-ethenoguanosine (**4**) and the simplest Y-nucleoside, wyosine (**1**). Therefore, a direct methylation in the 4-position of the tricylic etheno compound would give rise to a straightforward synthesis of the fluorescent Y-nucleosides, avoiding a laborious, multi-step procedure *via* 3-methylguanosine, elaborated by Itaya et al. (1980).

**Scheme 5:**
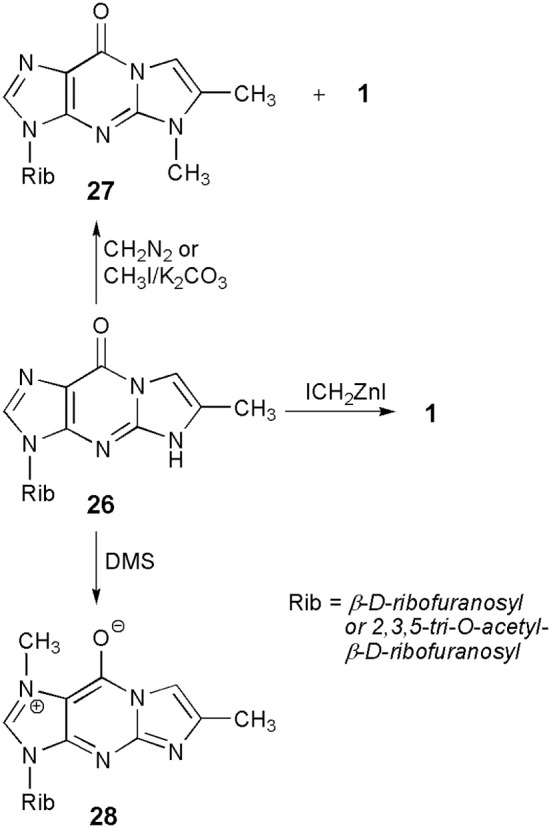
**Methylation of 4-desmethylwyosine**.

A common substrate for the methylation study—4-desmethylwyosine (i.e., 6-methyl-1,N^2^-ethenoguanosine; compound **26** in Scheme 5), may easily be obtained by treatment of guanosine (**6**) with bromoacetone (Kasai et al., 1976). Depending on the reaction conditions, the tricyclic system of **26** may be methylated in different positions. Thus, reaction with diazomethane (Kasai et al., 1976) or with methyl iodide, in the presence of potassium carbonate (Boryski and Ueda, 1985), leads to the 5-methyl product (**27**), a non-fluorescent isomer of wyosine. In turn, reaction with dimethyl sulfate (DMS) provides fluorescent 1,6-dimethyl-1,N^2^-ethenoguanosine (**28**), a tricyclic analog of 7-methylguanosine (Golankiewicz and Folkman, 1983). However, the same author, having carefully investigated the methylation of **26** triacetate with diazomethane, demonstrated that triacetate of **1** is formed as a minor product (3%) of this reaction, occurring in addition to predominant amounts of the 5-methyl product (**27**). However, a major breakthrough on the way to Y-nucleosides was the application of the Simmons-Smith organozinc reagent, ICH_2_ZnI (Bazin et al., 1987), which effectively transforms the 4-desmethylwyosine system (**26**) into wyosine (**1**; yield *ca*. 70%).

Besides N-alkylation reactions, some derivatives of 4-desmethylwyosine (**26**) undergo an unexpected C-7-aralkylation, as reported by Zeidler and Golankiewicz (1998), and Ostrowski et al. (2000, 2005). The aforementioned reaction takes place when a bulky substituent (e.g., phenyl) is present in the 6-position of substrates, and alkyl halides capable to form stable carbocations, like triphenylmethyl chloride or benzyl bromide, are applied.

### Ring-opening reactions

**Scheme 6:**
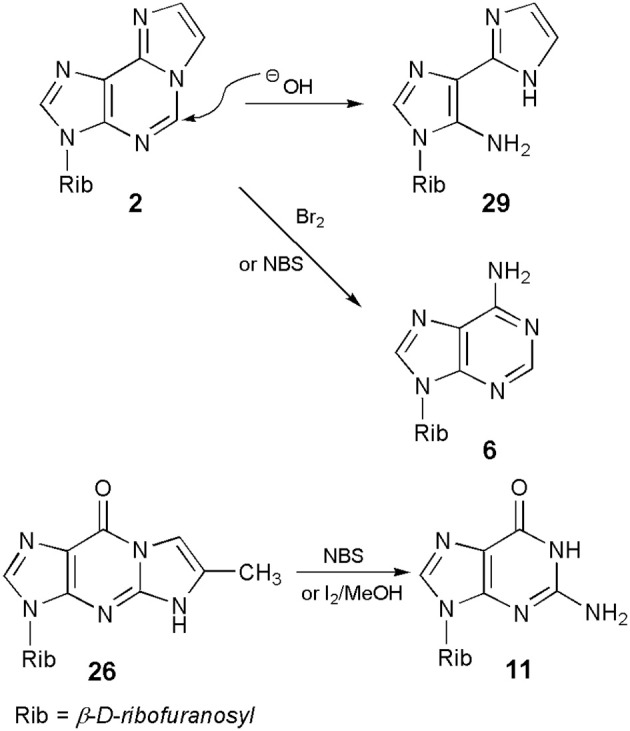
**Ring-opening reactions of ethenonucleosides**.

In general, ethenonucleosides are not very stable chemical species. It is evident in the case of 1,N^6^-ethenoadenosine (**2**), which upon treatment with 0.1 N sodium hydroxide at room temperature, is almost quantitatively transformed into a pyrimidine ring-opened compound, 3-β-d-ribofuranosyl-4-amino-(5-imidazo-2-yl)-imidazole (**29**; Scheme 6; Yip and Tsou, 1973). No mechanism has been proposed for this ring-opening reaction, but one may assume that it could be an initial nucleophilic attack of hydroxide anion at C5, followed by hydrolysis to **29**. The reaction is perhaps closely related to the well-known Dimroth rearrangement of adenine nucleosides. In turn, the additional etheno ring of **2** and its derivatives may be cleaved on bromination with Br_2_ or with N-bromosuccinimide (NBS), providing in this way a starting adenine nucleoside (Yamaji et al., 1977). To elucidate the reaction mechanism, the authors postulated a complicated sequence of events, with an initial bromination at either 7- and/or 8-position, and the final hydrolysis of brominated intermediates.

A similar removal of the etheno ring, under oxidizing condition, has been observed in the case of the linear 5-methyl-1,N^2^-ethenoguanosine (**26**) and its derivatives (Boryski and Ueda, 1985; Boryski et al., 1988a, 1996). Thus, treatment of ethenonucleosides of the general type **17** (including **26**) with NBS or iodine in methanol, followed by alkaline hydrolysis, provides the corresponding guanine nucleosides (type **11**) in good yields. We have not find any report on an analogous reaction of the angular N^2^,3-etheno compound (**5**), but a similar process of removing of the etheno bridge seems to be quite possible in that case.

Interestingly, the stability of 1,N^2^-ethenoguanosine derivatives depends on the kind of substituents in the 6-position. As shown by Amblard et al. (2009), there is a correlation between the stability and end electron effects of 6-aryl substituents in tricyclic nucleosides related to the linear ethenoguanosine. For example, a 6-(4-dialkylamino)phenyl acyclonucleoside undergoes fast decomposition in water, at room temperature (τ_1/2_ 25 min).

### Transglycosylation reactions

**Scheme 7:**
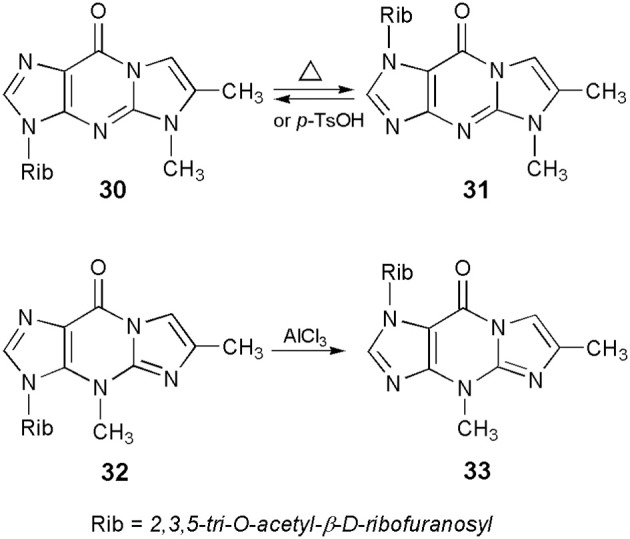
**Transglycosylation reactions of 5- and 4-methyl ethenoguanosines**.

Protected nucleosides, derived from the linear 1,N^2^-ethenoguanosine, like other 6-oxopurine nucleosides (Boryski, 2008), readily undergo transglycosylation reactions, i.e., a transfer of the sugar residue from one nitrogen atom to another. For example, crystalline 5,6-dimethyl-1,N^2^-ethenoguanosine triacetate (or 5-methyl-4-desmethylwyosine triacetate; compound **30** in Scheme 7), when heated without solvents at 200–230°C for a short period of time (5–10 min), isomerizes to a fluorescent 1-(2,3,5-tri-O-acetyl)-β-d-ribofuranosyl compound (**31**; Boryski, 1996). Similar results are observed in solvents, in the presence of acidic catalysts, e.g., boiling chlorobenzene and *p*-toluenesulfonic acid. The reaction is fully reversible—no matter which isomer (**30** or **31**) is used as a substrate, transglycosylation provides an almost equimolar mixture of both compounds (the 3-glycosyl isomer is usually slightly prevailing). In turn, wyosine triacetate (**32**) undergoes an irreversible isomerization to the corresponding 1-riboside (**33**), under very mild conditions (methylene chloride, 0°C, AlCl_3_ as a catalyst; Glemarec et al., 1988). This fact clearly reflects an unusual instability of the N-glycosidic bond in Y-nucleosides (Blobstein et al., 1975; Kasai et al., 1976).

Besides 1,N^2^-ethenoguanosine derivatives, the angular N^2^,3-ethenoguanosine nucleosides may probably undergo transglycosylation reactions, since they can be considered as 6-oxopurine nucleosides, but so far this has not been proven experimentally. Glycosyl migration reactions of other ethenonucleosides, derived from adenosine or cytidine, have not been reported in the literature.

## Application of etheno compounds

Since their discovery in the 1970's, ethenocleosides found a variety of applications in the structural studies of macromolecules, such as nucleic acids and proteins. Due to their fluorescence, 1,N^6^-ethenoadenosine (**2**) and 1,N^4^-ethenocytidine (**3**), as well as their 2′-deoxy and other derivatives, were applied as versatile probes to investigate mechanisms of enzymatic reaction and three-dimensional structure of RNAs and DNAs. Those rather historical applications have been described or reviewed in many papers (e.g., Secrist et al., 1972; Barrio et al., 1972; Steiner et al., 1973; Schulman and Pelka, 1976; Kost and Ivanov, 1980; Leonard, 1984). The formation of ethenonucleosides at the polynucleotide level has also been intensively studied for better understanding of the toxic influence of carcinogens on nucleic acids, and the mechanism of mutagenesis (e.g., Singer et al., 1987; Kusmierek and Singer, 1992; Golding et al., 1996; Gómez-Bombarelli et al., 2011; Bonnac et al., 2013; Calabretta and Leumann, 2013; Ogawa et al., 2013).

**Scheme 8:**
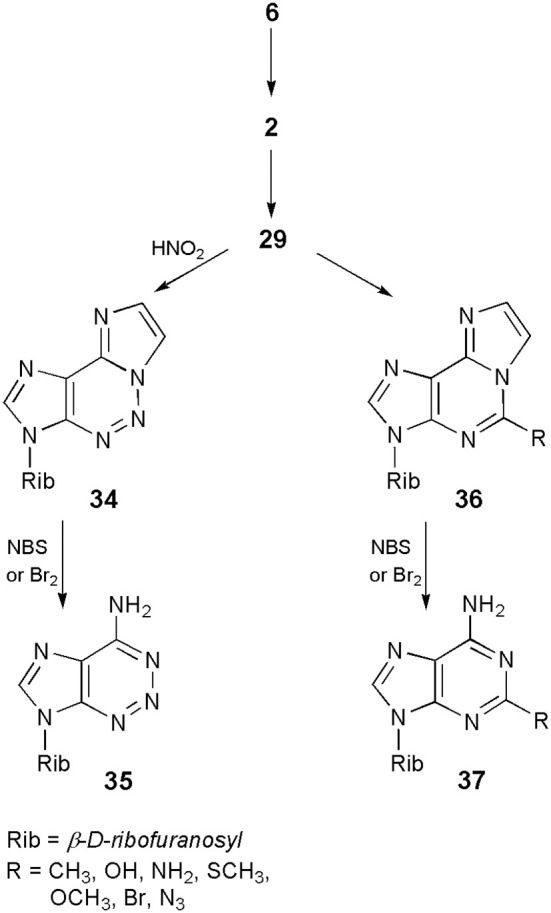
**Application of 1,N^6^-ethenoadenosine in the synthesis of 2-azaadenosine an 2-subtituted adenosine derivatives**.

On the other hand, what may turn out to be more beneficial for nucleoside chemists, the ethenonucleosides have found applications as useful synthons in obtaining a number of nucleoside analogs. The approach takes advantage of the fact that etheno modified compounds undergo different reactions than their parent nucleosides (*vide ante*). Thus, as shown in Scheme 8, adenosine (**6**), upon treatment with CAA, can easily be transformed into 1,N^6^-ethenoadenosine (**2**), which then undergoes a pyrimidine ring-opening reaction in 0.1 N sodium hydroxide to form 3-β-D-ribofuranosyl-4-amino-(5-imidazo-2-yl)-imidazole (**29**). The latter compound, treated with sodium nitrite in acetic acid, may be converted to a 5-aza analog of 1,N^6^-ethenoadenosine (**34**; Yip and Tsou, 1973). Finally, removal of the etheno ring on bromination with bromine or NBS produces 2-azaadenosine (**35**), in good overall yield (Yamaji and Kato, 1975). A similar sequence of ring-opening–ring-closure reactions has been applied by Yamaji et al. (1976, 1977), for the synthesis of a series of 5-substituted derivatives of 1,N^6^-ethenoadenosine (**36**), followed by deblocking to the respective 2-substituted adenosines (general structure **37**). An analogous approach however, would be useless in the case of 1,N^4^-ethenocytidine, due to the reactivity of its C7=C8 double bond, under bromination conditions.

**Scheme 9:**
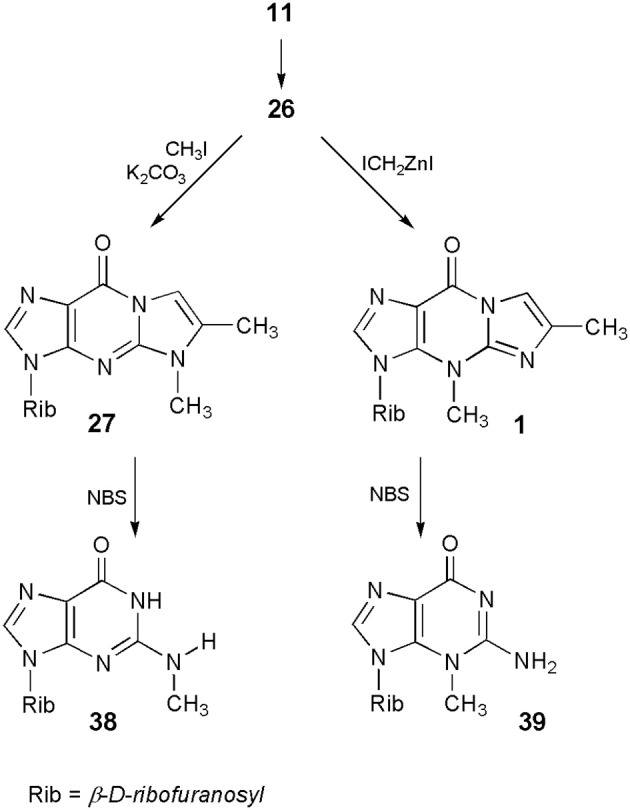
**Application of 4-desmethylwyosine in the synthesis of N^2^- and 3-methylguanosine**.

In turn, application of 6-methyl-1,N^2^-ethenoguanosine (**26**) makes it possible to perform the synthesis of either N^2^-methyl- or 3-methylguanosine, which cannot be obtained by direct methylation of the parent nucleoside. As presented in Scheme 9, guanosine (**11**) can be transformed into its tricylic derivatives (**26**) which, depending on the methylating reagent (*vide ante*) may give either 5-methyl (**27**) or 4-methyl (**1**) isomeric products. In the final step, deblocking of **27** with the use of NBS leads to N^2^-methylguanosine (**38**; Boryski and Ueda, 1985), while removal of the etheno ring, in the case of 4-methyl nucleoside (**1**), gives 3-methylguanosine (**39**; Boryski et al., 1988a).

**Figure 2:**
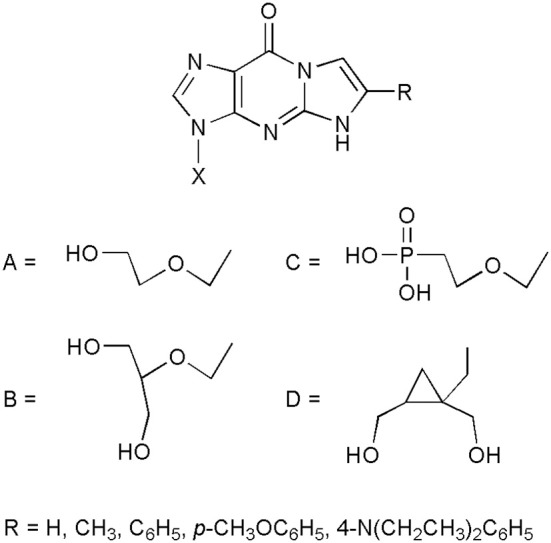
**Selected examples of antiviral acyclonucleosides derived from 1,N^2^-ethenoguanine**.

But perhaps the most spectacular application of etheno compounds has been found while searching for new antiviral nucleoside analogs. It has been demonstrated that a potent antiviral drug, acyclovir (ACV; Zovirax; 9-(2-hydroxyethoxymethyl)guanine; Elion et al., 1977) retains its antiherpetic activity after chemical modification of the 1,N^2^-etheno type (Boryski et al., 1988b, 1991). Some selected examples of bioactive ethenoguanines are presented in Figure 2. The first synthesized acyclonucleoside, containing an etheno ring, 1,N^2^-isopropenoacyclovir (TACV; X=A, R=CH_3_ Boryski et al., 1988b), has become a leading compound when it comes to searching for new antivirals of this type (Golankiewicz et al., 1991, 1994, 2001; Balzarini et al., 2002; Goslinski et al., 2002, 2003; Ostrowski et al., 2005, 2006, 2009; Horejsi et al., 2006).

As suggested by Amblard et al. (2009), ethenoguanine nucleosides probably act as prodrugs, releasing the active acyclonucleosides (i.e., acyclovir or other biologically active nucleoside analogs) after the hydrolysis of the etheno ring, under physiological conditions. Indeed, the antiviral activity is well-correlated with stability of tricyclic nucleosides. The most unstable analogs, equipped with 6-phenyl, substituted with electron donating groups, are the most active ones, reaching the level of activity of the parent nucleosidic drugs. Biological activity of the etheno acyclonucleosides has been described and discussed in our recent review (Jahnz-Wechmann et al., 2015), and in the previous review articles (Golankiewicz and Ostrowski, 2006; De Clercq, 2010).

## Concluding remarks

The ethenonucleosides, along with furano- and pyrrolopyrimidine nucleosides (Jahnz-Wechmann et al., 2015; and references cited herein), constitute the most important class of fused nucleosides, possessing a promising therapeutic potential. In the present review, we focused our attention on the relatively new literature data, concerning the ethenonucleosides, their synthesis, chemistry and applications. Unlike the previous papers, devoted mainly to derivatives of adenosine and cytidine (see Section Introduction), our paper presents many aspects of chemistry and activity of less known derivatives of ethenoguanosine.

The state of the art in the studies of etheno compounds, described herein, may suggest that everything in this field has already been done, and almost everything has been elucidated. However, at the end of this presentation, we would like to give some examples of problems awaiting solution. Firstly, it is still unknown whether the bioactive ethenonucleosides would be of any use in clinical treatment of viral infections. Can the lower toxicity and enhanced lipophilicity of etheno prodrugs compensate their generally lower activity? Secondly, many possible etheno prodrugs of bioactive adenosine and cytidine have not yet been obtained, and there are almost no reports on biological screening in this case. We do not know what is the real stability of ethenoadenosine and ethenocytidine derivatives under physiological conditions, which would be critical to determine their possible therapeutic applications. Moreover, the etheno modifications have not been reported for a number of modified nucleosides, like aza- and deaza-analogs, 8-substituted purine nucleosides, or derivatives of 2-aminopurine, which could form both angular and linear etheno isomers. Finally, the not yet studied ring opening reaction of the angular N^2^,3-ethenoguanosine may find interesting synthetic applications.

## Author contributions

ZJ, literature search, organization of references, final correction. GF, literature search, graphical art, final correction. PJ, literature search, critical analysis of data. JB, general conception, writing the manuscript, coordination of the team work.

## Funding

The financial support from the Polish Ministry of Science and Higher Education under statutory financing and the KNOW program is gratefully acknowledged.

### Conflict of interest statement

The authors declare that the research was conducted in the absence of any commercial or financial relationships that could be construed as a potential conflict of interest.
